# Human α1-Antitrypsin Binds to Heat-Shock Protein gp96 and Protects from Endogenous gp96-Mediated Injury *In vivo*

**DOI:** 10.3389/fimmu.2013.00320

**Published:** 2013-10-28

**Authors:** David E. Ochayon, Mark Mizrahi, Galit Shahaf, Boris M. Baranovski, Eli C. Lewis

**Affiliations:** ^1^Department of Clinical Biochemistry and Pharmacology, Faculty of Health Sciences, Ben-Gurion University of the Negev, Be’er Sheva, Israel

**Keywords:** heat-shock protein, inflammation, pancreatic islets, diabetes, α1-antitrypsin, allogeneic transplantation

## Abstract

The extracellular form of the abundant heat-shock protein, gp96, is involved in human autoimmune pathologies. In patients with type 1 diabetes, circulating gp96 is found to be elevated, and is bound to the acute-phase protein, α1-antitrypsin (AAT). The two molecules also engage intracellularly during the physiological folding of AAT. AAT therapy promotes pancreatic islet survival in both transplantation and autoimmune diabetes models, and several clinical trials are currently examining AAT therapy for individuals with type 1 diabetes. However, its mechanism of action is yet unknown. Here, we examine whether the protective activity of AAT is related to binding of extracellular gp96. Primary mouse islets, macrophages, and dendritic cells were added recombinant gp96 in the presence of clinical-grade human AAT (hAAT, Glassia™, Kamada Ltd., Israel). Islet function was evaluated by insulin release. The effect of hAAT on IL-1β/IFNγ-induced gp96 cell-surface levels was also evaluated. *In vivo*, skin transplantation was performed for examination of robust immune responses, and systemic inflammation was induced by cecal puncture. Endogenous gp96 was inhibited by gp96-inhibitory peptide (gp96i, Compugen Ltd., Israel) in an allogeneic islet transplantation model. Our findings indicate that hAAT binds to gp96 and diminishes gp96-induced inflammatory responses; e.g., hAAT-treated gp96-stimulated islets released less pro-inflammatory cytokines (IL-1β by 6.16-fold and TNFα by 2.69-fold) and regained gp96-disrupted insulin release. hAAT reduced cell activation during both skin transplantation and systemic inflammation, as well as lowered inducible surface levels of gp96 on immune cells. Finally, inhibition of gp96 significantly improved immediate islet graft function. These results suggest that hAAT is a regulator of gp96-mediated inflammatory responses, an increasingly appreciated endogenous damage response with relevance to human pathologies that are exacerbated by tissue injury.

## Introduction

Pancreatic islet transplantation can afford tight physiological blood glucose control for individuals with type 1 diabetes, provided that the grafted islets survive multiple inflammatory, innate, and immune-mediated injurious processes. During the initial stages of islet engraftment, prior to lymphocytic antigen recognition, macrophages and dendritic cells amplify the inflammatory state of graft microenvironment, causing the loss of up to 60% of the transplanted islet mass (1). The trigger for cell activation falls under the category of sterile inflammation, instigated by injured and necrotic isolation-processed islets. Indeed, it has been shown that inflammation from damaged foci readily spreads to neighboring islets independent of adaptive immune responses; these neighboring islets in turn become new foci of inflammation (2). Current treatment protocols that accompany islet transplantation predominantly target adaptive immune responses and exhibit limited outcomes, as well as pose a direct threat to islet β-cell viability (3–5); inflammation, on the other hand, is left primarily unopposed, as corticosteroid therapy is contraindicated (6).

Upstream events that govern sterile inflammation involve endogenous alarmin molecules, or damage associated molecular pattern (DAMP) molecules (7, 8). For example, heat-shock protein gp96 is an abundant chaperone in the endoplasmic reticulum in a wide variety of mammalian cells, and its intracellular levels rise appropriately during stress conditions such as inflammation, hypoxia, or glucose starvation (9). However, under certain conditions, gp96 is found outside the cells and on the membranes of some immune cells. Recently, evidence for aberrant extracellular functions of gp96 have emerged, suggesting that once exiting the cells, gp96 behaves as a DAMP molecule.

Extracellular gp96 binds to TLR2/4 and to CD91, which in turn facilitate an increase in the release of pro-inflammatory cytokines (10, 11). Extracellular gp96 is obtained by both passive release from leaky cell membranes of affected necrotic cells, and by production of an inducible membrane-bound form under particular conditions (12, 13). In mice genetically engineered to possess antigen-presenting cells (APCs) that express gp96 on their membranes, systemic lupus erythematosus (SLE) symptoms are observed (12). Accordingly, APCs collected from these mice release high levels of pro-inflammatory mediators. In humans, Pope et al. described high levels of gp96 in synovial fluids from patients with rheumatoid arthritis (RA) compared to samples obtained from patients with osteoarthritis; the collected synovial gp96 was then shown to evoke an inflammatory response in human macrophages in the form of elevated *TLR2*, IL-6, and TNFα. Consistent with these findings, patients with type 1 diabetes exhibit high levels of circulating gp96, supporting the increasing notion that there exists a role for DAMPs in autoimmune conditions (14).

Extracellular gp96 has been detected in the circulation of patients with type 1 diabetes bound to the acute-phase protein α1-antitrypsin (AAT) (14). AAT is an anti-inflammatory protein that circulates at >1 mg/ml, second only to albumin and immunoglobulins in its levels. AAT is readily available for the treatment of the rare genetic deficiency in the protein, for which the material is provided in the form of plasma-derived affinity-purified human AAT (hAAT).

In recent years, several activities were observed that render hAAT an attractive candidate for a series of inflammatory conditions, as recently reviewed (15). In particular, a benefit appears to emerge in conditions that combine tissue damage and inflammation, such as in ischemic myocardium (16) and graft-versus-host disease (17). Evidence is most abundant, however, in the ability of hAAT to modify the adaptive immune response in favor of pancreatic islet survival; hAAT exerts protective activities in the context of autoimmune diabetes and islet alloimmune responses (18–22), and three independent trials are currently ongoing for hAAT therapy in newly diagnosed type 1 diabetes patients (NIH clinical trial registry NCT01304537, NCT01319331, and NCT01183468). Our group has demonstrated that hAAT also facilitates immune tolerance in several non-diabetes-related models (23, 24) and that hAAT affords strain-specific immune tolerance by targeting non-T cells. Indeed, hAAT appears to target predominantly APCs, as macrophages and dendritic cells.

In the present study, we examine whether the protective activities of hAAT may be derived from direct inhibition of gp96-mediated inflammatory responses, in the context of pancreatic islet transplantation.

## Materials and Methods

### Animals

Six to eight-week-old male C57BL/6 mice (Harlan Laboratories Ltd., Israel) were used as graft recipients and participated in the cecal puncture (CP) model. Pancreatic islets were isolated from 8- to 10-week-old CBA/2J mice (Jackson laboratory, Bar Harbor, ME, USA). Skin grafts were obtained from 8- to 10-week-old BALB/c mice (Harlan Laboratories Ltd.). Experiments were approved by the Ben-Gurion University of the Negev Animal Care and Use Committee.

### Pancreatic islet isolation

Pancreatic islets were isolated as described (20). Briefly, donor mice were anesthetized, pancreata were inflated with collagenase (1 mg/ml, type XI, Sigma-Aldrich), excised, and incubated for 40 min at 37°C. Digested pancreata were vortexed and filtered through a 500-μm sieve, and the pellet was washed in HBSS containing 0.5% BSA (Sigma-Aldrich). The pellet was resuspended in RPMI 1640 medium supplemented with 10% FCS, 50 units/ml penicillin, and 50 μg/ml streptomycin (all from Cellgro, Mediatech, Herndon, VA, USA). Islets were collected on a 100-μm cell strainer (BD, Falcon) and then hand-picked under a stereomicroscope.

### Islet allograft transplantation

Recipient mice were rendered hyperglycemic by single streptozotocin injection (STZ, 225 mg/kg i.p., Sigma-Aldrich), and islets were grafted under the renal capsule, as described (20). Briefly, hyperglycemic recipient mice were anesthetized, and 500 islets were released into the renal subcapsular space, which was immediately sealed with 1-mm^3^ sterile absorbable gelatin sponge (Surgifoam, Ethicon, Somerville, NJ, USA). Blood glucose levels were determined daily from tail blood using a standard glucometer (Roche).

### Histology and immunohistochemistry

Immunohistochemistry staining was performed as described (25). Briefly, explanted kidneys carrying implants were fixed in 10% formalin (Sigma-Aldrich) for 24 h and transferred into 70% ethanol. The specimens were cut through the center of the implant, embedded in paraffin, and sectioned. For histological examination, Hematoxylin and Eosin (H&E) was performed. Insulin immunostaining was performed using guinea-pig-anti-swine-insulin, detected by Cy3-donkey-anti-guinea-pig (both 1:200, DakoCytomation, Glostrup, Denmark); nuclei were depicted by 4′,6-diamidino-2-phenylindole (DAPI) staining (1 μg/ml, Sigma-Aldrich). Immunofluorescence was detected using Olympus BX60 (Olympus UK Ltd., London, UK).

### Gp96-related reagents

Recombinant human gp96 was from Proteintech, IL, USA. Endotoxin content was below 0.125 EU/ml at the concentrations used (LAL assay, Pyrotell^®^, East Falmouth, MA, USA). In addition, the recombinant protein was pretreated with PolymixenB (Sigma-Aldrich). Inhibitor of gp96 (gp96i; 37 amino-acid-long synthetic peptide II) was obtained from Compugen Ltd., Israel, described elsewhere (26).

### Macrophage activation study

Thioglycolate (3% v/v 72 h)-elicited peritoneal macrophages were collected by lavage and then further enriched using CD11b MACS bead-enrichment kit. Cells were then seeded (4 × 10^5^ per well in 48-well plate or 2 × 10^6^ per well in 6-well plate) in RPMI 1640 medium supplemented with 5% FCS, 50 units/ml penicillin, and 50 μg/ml streptomycin, in the presence or absence of various concentrations of recombinant gp96.

#### Cytokine analysis

Supernatants were collected 24 h later and analyzed by Q-Plex mouse cytokine chemiluminescence-based 8-p ELISA (Quansys Biosciences, Logan, UT, USA). Each cytokine was quantified by densitometry using Quansys Q-View software (Quansys Biosciences).

#### FACS analysis

Macrophages (1 × 10^6^ per sample) were stained with a combination of anti-CD11b-Pacific Blue, anti-F4/80-APC and anti-MHCII-APC/Cy7, anti-CD40-PE/Cy7, or anti-TLR4-PE/Cy7, and anti-TLR2-FITC (all antibodies obtained from Biolegend^®^). Antibodies were diluted to recommended concentration according to manufacturer’s instructions. Non-specific staining was excluded using matching isotype control antibodies (Biolegend^®^). Non-specific Fc staining was minimized by addition of Fc-blocking antibodies. Samples were analyzed by FACScalibur and BD Canto II; BD Biosciences. Data were analyzed by BD CellQuest Pro or FlowJo (version 7.6.5; Tree Star, Ashland, OR, USA).

### Cecal puncture model

A modification was made to the cecal and ligation model (27) with the aim of achieving a non-lethal tissue-damage-associated inflammatory model. Briefly, animals were anesthetized, the cecum was identified, and then punctured once with a 21-gage needle. A 1 mm-long section of feces was extruded, and the abdominal incision was closed using 3-0 nylon suture. Unlike in the complete ligation model, all mice survive the modified procedure. Recombinant gp96 (80 μg/kg) was introduced i.p. 24 h after CP, peritoneal lavage was performed 72 h after CP and then peritoneal cells were analyzed by FACS.

### Gp96 activity assays *in vitro*

#### Islet cultures

Thirty islets per well in quadruplicate were stimulated with 50 pM recombinant gp96 in the absence or presence of 3.3 μM gp96i. Supernatants were collected 24 h later for analysis by Q-Plex mouse cytokine chemiluminescence-based 8-p ELISA. Each cytokine was quantified by densitometry using Quansys Q-View software. Nitric oxide levels were evaluated by Griess reagent assay (Promega, WI, USA). Insulin levels were determined using specific ELISA (Mercodia, Sweden).

#### Macrophage-islet co-cultures

Thirty islets were cultured in quadruplicate in the presence or absence of primary macrophages (4 × 10^5^), with or without recombinant gp96 and treated in the presence or absence of gp96i at indicated concentrations.

### Bone marrow derived dendritic cells

Bone marrow derived dendritic cells (BMDC) were generated from mice as described (28). Briefly, bone marrow from the femurs and tibiae of C57BL/6 mice were washed in HBSS and cultured on 100 mm plates in batches of 2 × 10^6^ BM cells per plate in R10F (RPMI 1640 medium containing 2 mM l-glutamine, 50 units/ml penicillin, 50 μg/ml streptomycin, and 10% heat-inactivated FBS) with the addition of fresh 50 μM β2-mercaptoethanol and 20 ng/ml recombinant mouse GMCSF (Prospec, Israel).

### hAAT activity assays

#### Acellular gp96:hAAT binding assay

ELISA-plate wells were coated with gp96 (0.05–4 μg/ml) for 16 h at room temp. Blocking of non-specific interactions was achieved by incubation with 3% BSA solution (Sigma-Aldrich) for 2 h. Interaction between gp96 and hAAT (Glassia™, Kamada Ltd., Israel, 1 μg/ml) was allowed to occur for 2 h and hAAT detection was performed by HRP-conjugated anti-hAAT antibody (ICL, Portland, OR, USA).

#### hAAT binding to cell membrane

Peritoneal macrophages (2 × 10^6^ per well) were introduced to hAAT (0.5 mg/ml) for 16 h. Cells were stained with a combination of anti-F4/80-APC and anti-hAAT-FITC (Bethyl, Montgomery, TX, USA) and analyzed by FACS.

### Skin transplantation model

Allogeneic skin transplantation was performed between major MHC disparate mouse strains in order to evoke a potent and readily detectable immune response (skin donors: BALB/c, H-2^d^; skin recipients: C57BL/6, H-2^b^). Briefly, uniform 1 mm^3^ skin grafts were fixed into the inside wall of the peritoneal cavity of recipient mice, and the immune infiltrate was recovered by lavage and analyzed.

### *In vivo* gp96i treatment protocol

Mice were either injected with a daily dose of gp96i (4 mg/kg/day) dissolved in PBS (Biological industries, Israel) for 7 days, or were introduced with a 14-day osmotic pump containing a total of 1.4 mg gp96i (Alzet^®^, Cupertino, CA, USA), which distributed a continuous dose once implanted, equivalent to the injected 7-day protocol of 100 μg/day. The 7-day experiment was followed by animal harvest and splenocyte analysis for which cells were cultured on a 48-well plate (4 × 10^5^ cells per well), then supernatants were collected after 24 h, and analyzed by Q-Plex mouse cytokines chemiluminescence-based 14-p ELISA.

### Statistics

Comparisons between groups were performed by two-tailed Mann-Whitney U test or using Student’s *t*-test *p* < 0.05 was considered statistically significant. Statistical analysis was performed with GraphPad Prism 5.0 software.

## Results

### Inflammatory responses induced by gp96 are regulated by hAAT *in vitro*

**Figure 1:**
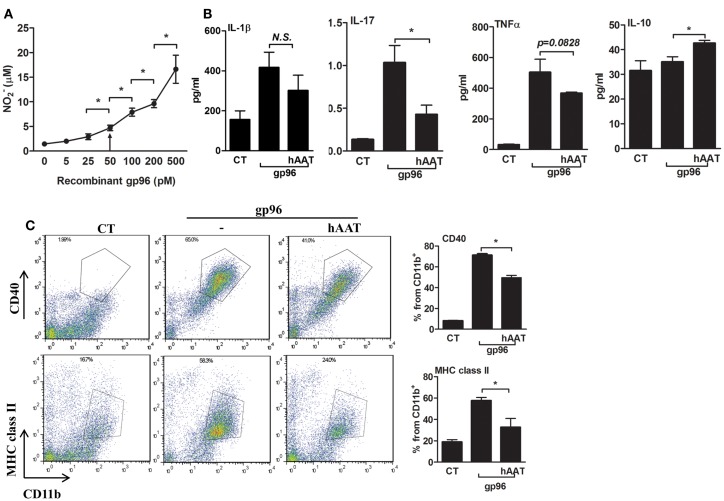
**Exogenous gp96 mediates inflammatory responses in peritoneal macrophages**. **(A)** Dose response curve. Peritoneal macrophages (4 × 10^5^ per well in quadruplicates) were incubated in the presence of gp96 (0–500 pM, indicated) for 24 h. Nitrile release to supernatants was evaluated by Griess assay. Shown are representative results of four independent experiments. **(B)** Release of inflammatory mediators. Peritoneal macrophages (4 × 10^5^ per well in quadruplicates) pretreated with human AAT (0.5 mg/ml) were incubated with recombinant gp96 (50 pM) for 48 h. Inflammatory mediators contained in the supernatants were measured by multiplex ELISA. CT, untreated cells. **(C)** Activation markers. Peritoneal macrophages (2 × 10^6^ per well in quadruplicates) pretreated with human AAT (0.5 mg/ml) were stimulated with recombinant gp96 (50 pM) for 48 h. Cells were then stained for surface markers. CT, untreated cells. Representative FACS dot plots, pooled results from three independent experiments. Mean ± SEM, *p < 0.05.

In order to determine whether exogenous gp96 induces an inflammatory response in murine macrophages, peritoneal macrophages were stimulated with several concentrations of polymixen B-pretreated recombinant gp96 (5–500 pM) followed by nitrile release assay 24 h later. As shown in Figure 1A, exogenous gp96 exerted an inflammatory response in a dose dependent manner, and a significant increase in nitrile release was observed at 50 pM and higher.

We next examined whether hAAT might reduce the inflammatory responses mediated by gp96 in peritoneal macrophages. Cell cultures were pretreated with or without hAAT (0.5 mg/ml, Glassia™, Kamada Ltd., Israel) overnight, then introduced to gp96 (50 pM) for 48 h. The inflammatory response was evaluated by cytokine accumulation and by surface expression of immune cell activation markers. As shown in Figure 1B, gp96 increased the release of inflammatory mediators (IL-1β 2.67 ± 0.5-fold, IL-17 7.6 ± 0.06-fold, and TNFα 15.94 ± 0.36-fold, compared to control untreated cells). In contrast, cultures that were stimulated by gp96 in the presence of hAAT displayed a decrease in the release of pro-inflammatory cytokines (IL-1β by 1.38 ± 0.18, IL-17 by 2.41 ± 0.1, and TNFα 1.36 ± 0.01, compared to gp96-stimulated cells); additionally, a rise was detected in the anti-inflammatory cytokine, IL-10 (1.21 ± 0.03-fold from gp96-stimulated). Surface markers are depicted in Figure 1C. As shown, gp96 elicited an increase in surface CD40 (8.78 ± 0.15-fold) and MHC class II (3.05 ± 0.15-fold) compared to untreated control cells. On the other hand, hAAT-treated cultures displayed lower surface levels of MHC class II and CD40 (1.61 ± 0.14-fold and 1.43 ± 0.03-fold, respectively, *p* < 0.05 for both).

### hAAT diminishes gp96-dependent inflammation *in vivo*

**Figure 2:**
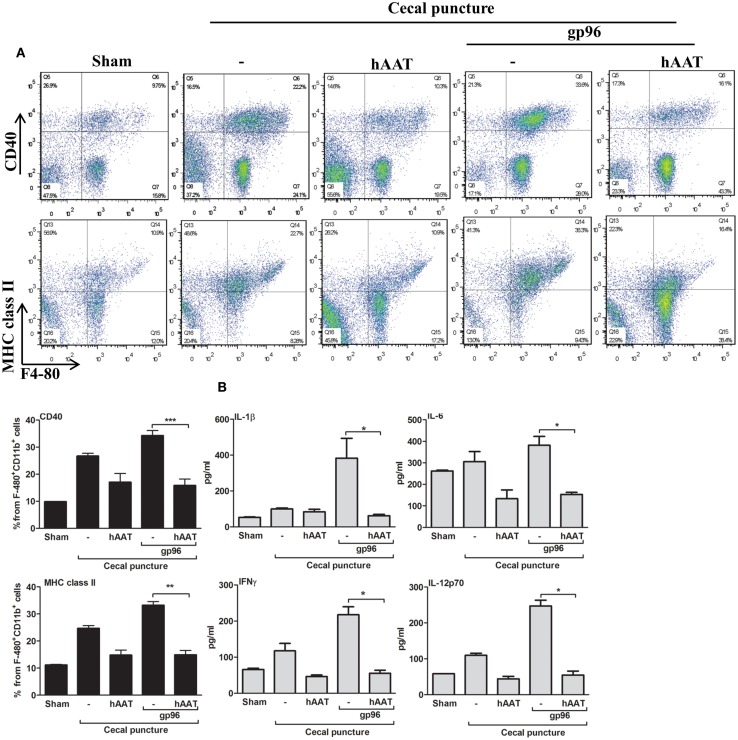
**The effect of hAAT on inflammatory responses during the cecal puncture (CP) model**. CP was performed by inflicting a uniform-size at a constant position of the cecum in mice that were divided into the following groups: untreated sham-operated mice (Sham, *n* = 2), untreated CP-operated mice (*n* = 4), mice pretreated with hAAT (60 mg/kg), and then CP-operated (*n* = 5), and CP-operated mice that were also introduced with recombinant gp96 (80 μg/kg i.p., once, 24 h after the surgical procedure). **(A)** Peritoneal cells. Cells were collected from the peritoneal cavity by lavage 72 after CP and immediately stained for surface activation markers. Representative dot plots are shown. *Bottom*, pooled results are presented. **(B)** Serum cytokines. Cytokine concentrations in the serum were evaluated 72 h after CP using multiplex ELISA. Representative results out of two independent experiments. Mean ± SEM, *p < 0.05, **p < 0.01, ***p < 0.001.

Cecal puncture, a modification of the sepsis model cecal ligation and puncture, evokes non-lethal systemic inflammation. Here, experimental groups included sham-operated mice, CP in the presence or absence of hAAT (60 mg/kg i.p., 24 h prior to CP) and CP in the presence or absence of hAAT with an added single dose of recombinant gp96 (80 μg/kg, 24 h after CP for the purpose of further aggravation of the inflammatory response in a gp96-dependent manner). At 72 h from CP, lavage was performed and recovered peritoneal cells were analyzed by FACS; surface levels of CD40 and MHC class II on F4-80^+^/CD11b^+^ cells are shown in Figure 2A.

**Figure A1:**
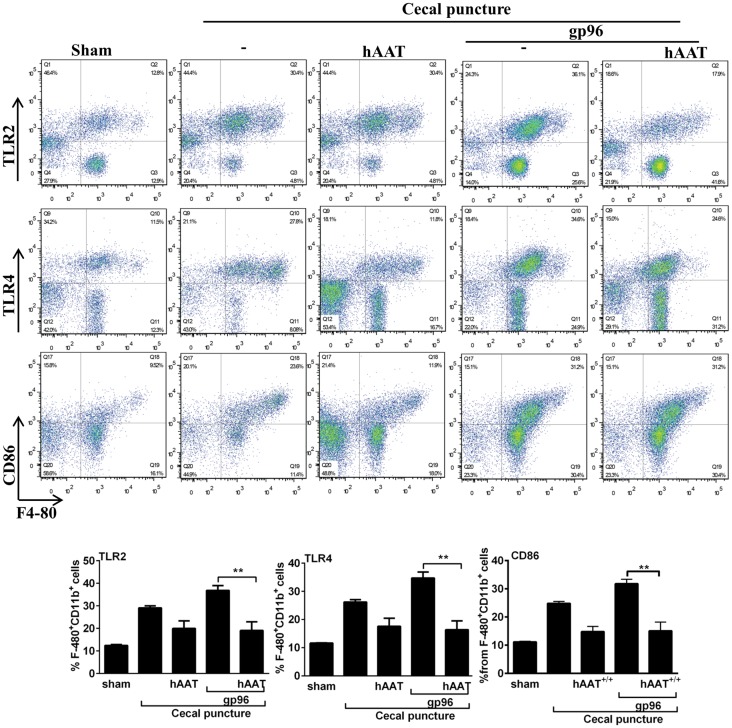
**The effect of hAAT on TLR2 and TLR4 during cecal puncture (CP) model**. CP was performed with the following groups: untreated sham-operated mice (Sham, *n* = 2), untreated CP-operated mice (*n* = 4), mice pretreated with hAAT (60 mg/kg), and then CP-operated (*n* = 5). Also, CP-treated mice were introduced recombinant gp96 (i.p., 80 μg/kg, once, 24 h after the surgical procedure). Peritoneal cells were obtained 72 after CP by lavage and immediately stained for surface activation markers. Representative dot plots are shown. *Bottom*, pooled results. Representative results out of two independent experiments. Mean ± SEM, *p < 0.05, **p < 0.01, ***p < 0.001.

As shown, CP significantly elevated surface expression of MHC class II and CD40 in comparison to sham-operated mice. Moreover, a significant increase in the expression of TLR2, TLR4, and CD86 was observed (Figure A1 in Appendix). The inflammatory phenotype of CP-treated mice was exacerbated by the addition of exogenous gp96 (CD40 and MHC class II rose by 1.28 ± 0.07-fold and by 1.34 ± 0.05-fold, respectively, compared to the CP group). In contrast, treatment with hAAT reduced surface expression levels of MHC class II and CD40 in CP-operated mice, and demonstrated a similar trend in mice treated with systemic gp96, as illustrated by reduction of MHC class II and CD40 to near-background levels.

Serum cytokine levels were determined at 72 h from CP (Figure 2B) and appeared to reflect the changes observed in cell-surface markers. For example, the addition of exogenous systemic gp96 to CP-operated mice elevated IL-1β 3.84 ± 1.11-fold, IFNγ 1.84 ± 0.25-fold, IL-6 1.24 ± 0.13-fold, and IL-12p70 2.25 ± 0.14-fold compared to the CP group. In contrast, hAAT-treated mice demonstrated a significant decrease in pro-inflammatory serum cytokine levels.

### hAAT diminishes gp96-driven islet and BMDC inflammation *in vitro*

**Figure 3:**
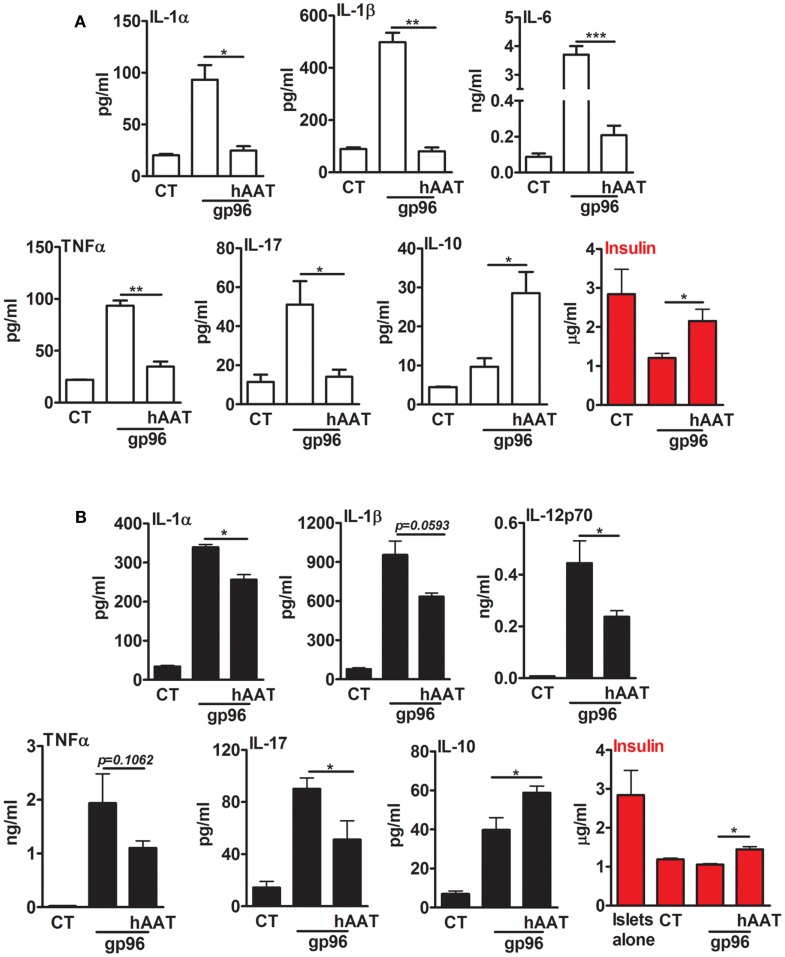
**Exogenous gp96 mediates inflammatory responses and impairs insulin release from pancreatic islets**. Primary mouse islets (30 per well in triplicates) were incubated in the presence or absence of BMDCs (4 × 10^5^ per well) that were pretreated with human AAT (0.5 mg/ml). Cells were incubated with recombinant gp96 (50 pM) for 48 h. Inflammatory mediators and insulin levels were determined by multiplex ELISA and standard ELISA, respectively. CT, untreated cells. **(A)** Islets, **(B)** co-culture; representative results from four independent experiments. Mean ± SEM, *p < 0.05, **p < 0.01, ***p < 0.001.

In a simplified representation of an islet graft microenvironment, gp96 was added to resting primary mouse pancreatic islets (Figure 3A) and to a mixed co-culture of islets and BMDCs (Figure 3B) for 48 h. Cell cultures were pretreated with hAAT or medium overnight. The inflammatory response was evaluated by cytokine accumulation, and islet viability was evaluated by accumulated insulin. As shown in Figure 3A, cultured islets introduced to gp96 displayed increased release of inflammatory mediators (IL-1α increased 4.34 ± 3.2-fold, IL-1β 5.05 ± 0.01-fold, IL-6 42.4 ± 0.004-fold, TNFα 4.22 ± 0.002-fold, and IL-17 4.41 ± 0.9-fold, compared to resting islets). Accordingly, under these conditions, islets displayed loss of function in the form of reduced insulin release (2.35 ± 0.04-fold lower levels in comparison to resting islets). In contrast, in the presence of hAAT, islets exposed to gp96 demonstrated a decrease in inflammatory mediators (IL-1α by 1.64 ± 0.23-fold, IL-1β 6.16 ± 0.02-fold, IL-6 14.76 ± 0.013-fold, TNFα 2.69 ± 0.05-fold, and IL-17 3.61 ± 0.07-fold, compared to gp96-treated islets); islets also appeared to have partially retained insulin release, compared to gp96-treated islets.

A similar trend was observed in a mixed co-culture containing BMDCs and islets (Figure 3B). Introduction of gp96 to co-cultures resulted in an increase in the release of inflammatory mediators (IL-1α 9.95 ± 0.21-fold, IL-1β 12.05 ± 1.12-fold, IL-12p70 63.73 ± 12.33-fold, TNFα 88.92 ± 25.71-fold, and IL-17 6.3 ± 0.58-fold, compared to resting islets); this was accompanied by a decrease in accumulated insulin levels. On the other hand, in the presence of hAAT, a decrease in the release of inflammatory mediators was observed (IL-1α 1.32 ± 0.03-fold, IL-1β 1.48 ± 0.02-fold, IL-12p70 1.87 ± 0.04-fold, TNFα 1.75 ± 0.06-fold, and IL-17 1.75 ± 0.15-fold, compared to co-culture introduced to gp96). In addition, in the presence of hAAT, an elevation in IL-10 was observed (1.48 ± 0.08-fold increase, compared to gp96-treated cells). Moreover, hAAT-treated co-cultures retained insulin release in comparison to gp96-treated co-cultures.

### Membrane-bound gp96 is elevated during inflammation and binds hAAT

**Figure 4:**
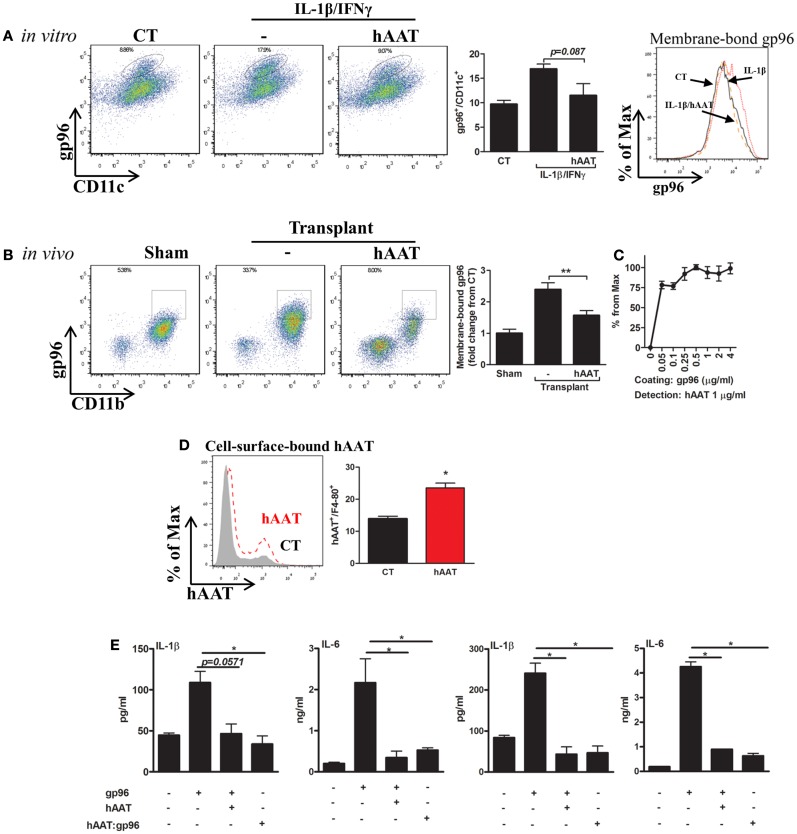
**hAAT regulates membrane-bound gp96 levels under inflammatory conditions *in vitro* and after allogeneic skin transplantation *in vivo*; direct binding of hAAT to gp96 on cell membranes**. **(A)** Cell culture study. Primary BMDCs (2 × 10^6^ per well) were pretreated with human AAT (0.5 mg/ml) and stimulated with IL-1β combined with IFNγ (5 ng/ml each) for 48 h. CT, untreated cells. *Left*, representative FACS analysis dot plots and pooled results from three independent experiments. *Right*, representative histograms of CD11c-positive cells. **(B)** Skin allograft model. Skin from BALB/c mice (H-2^d^) was stitched into the inner side of the peritoneal cavity wall of C57BL/6 mice (H-2^b^). Mice were either untreated or pretreated (hAAT, 60 mg/kg). Seventy-two hours after transplantation, lavage was performed and collected cells were examined for levels of membrane-bound gp96 by FACS analysis. Representative dot plots and pooled results out of two independent experiments. **(C)** Non-cellular binding assay. ELISA-plate wells were coated with gp96 (0–4 μg/ml) for 16 h and then incubated for 3 h with hAAT (1 ng/ml). Captured hAAT was determined by HRP-conjugated anti-hAAT antibody. **(D)** Cell membrane-bound hAAT. Primary peritoneal macrophages (2 × 10^6^ per well in quadruplicates) were incubated with hAAT (0.5 mg/ml) for 16 h and then stained with anti-hAAT antibody. hAAT binding to cell membranes was evaluated by FACS. CT, untreated cells. Representative results out of three independent experiments. **(E)** Pre-complexed hAAT:gp96. Freshly isolated primary mouse islets (30 per well in triplicates) or macrophages (4 × 10^5^ per well in quadruplicates) were incubated with recombinant gp96 (50 pM) in the presence or absence of hAAT (0.5 mg/ml) or incubated with a complex of hAAT and gp96 (hAAT:gp96 at 0.5 mg/ml and 50 pM, respectively, for 1 h). Cells were analyzed 48 h later. CT, untreated cells. Representative results from three independent experiments. Mean ± SEM, *p < 0.05, **p < 0.01.

Membrane-bound gp96 was readily detected in both CD11b^+^ cells and CD11c^+^ cells in a cytokine-stimulation assay *in vitro* (Figure 4A) and in a skin transplantation model *in vivo* (Figure 4B). In both setups, hAAT was assessed for its effects on membrane-bound gp96 levels, as well as its association with gp96.

Bone marrow derived dendritic cells were pretreated with hAAT or medium overnight, then incubated in the presence of IL-1β and IFNγ (5 ng/ml each) for 24 h. Cells positive for surface gp96 were determined by direct FACS analysis using anti-gp96 PE-conjugated antibody. As shown in Figure 4A, in the presence of IL-1β and IFNγ stimulation, cell-surface gp96 was increased in BMDCs 1.74 ± 0.1-fold, compared to non-stimulated cells. However, hAAT-treated stimulated cultures exhibited a reduction in membrane-bound gp96 (1.47 ± 0.14-fold from stimulated cells). The possibility of masking gp96 from its detection-antibody by the presence of hAAT was excluded in separate experiments, where hAAT was directly mixed into the FACS staining buffer (not shown).

Membrane-bound gp96 was examined *in vivo* in a skin allograft transplantation model performed in the peritoneal cavity for the purpose of improved access to infiltrating cell retrieval. Peritoneal lavage was performed 72 h later and CD11b^+^/F4-80^+^ cells were analyzed for relative surface gp96 levels (Figure 4B). Groups included sham-operated mice, and mice injected with hAAT or PBS 24 h prior to skin transplantation. As shown in Figure 4B, grafted WT mice demonstrated a 2.39 ± 0.21-fold increase in surface expression of gp96, compared to sham-operated mice. In contrast, hAAT-treated mice exhibited lower surface expression of gp96, compared to PBS-treated grafted mice (1.52 ± 0.06-fold).

Direct binding between gp96 and hAAT was demonstrated: in a cell-free assay (Figure 4C), cell-surface binding assay (Figure 4D), and in a complexed form (Figure 4E). As shown, binding of hAAT to gp96-coated wells depicted a robust binding capacity across several orders of concentrations (hAAT:gp96 molar ratio range: 0.1–8.0) (Figure 4C). Binding to cell membrane was evaluated using peritoneal macrophages that were incubated in the presence or absence of hAAT. As shown in Figure 4D, hAAT displayed direct binding to the cell surface. Finally, pancreatic islets and macrophage cultures introduced to hAAT that was pre-incubated with gp96 displayed a similar immune response to hAAT-treated cultures, hence a neutralization of exogenous gp96 (Figure 4E).

### Inhibition studies using gp96i depict a role for endogenous gp96 in cell stimulation and immediate loss of islet graft function

**Figure 5:**
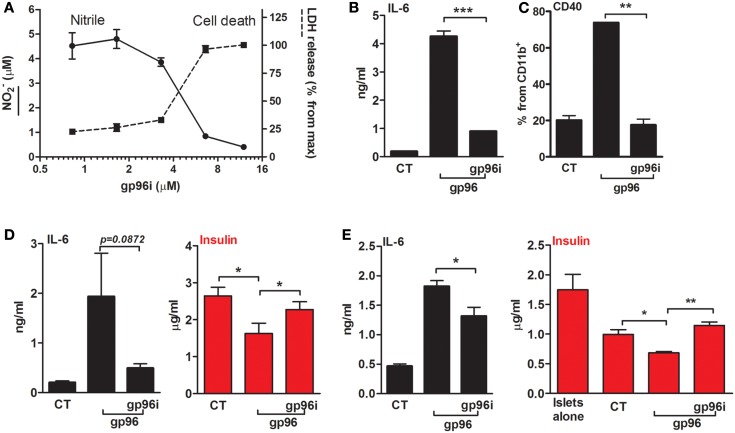
**An inhibitor of gp96 (gp96i) modulates inflammatory responses in macrophages and islet cultures**. **(A)** Calibration assay. Peritoneal macrophages (4 × 10^5^ per well in quadruplicates) were incubated in the presence of recombinant gp96 (50 pM) and gp96i (0–16 μM) for 24 h. Nitrile release into supernatants was evaluated by Griess assay. Cell death was evaluated by LDH release into the supernatants, normalized to the output of maximal gp96i concentration. **(B)** Release of inflammatory mediators. Peritoneal macrophages (4 × 10^5^ per well in quadruplicates) were pretreated with gp96i (3.3 μM) and then incubated with recombinant gp96 (50 pM) for 48 h. IL-6 levels in supernatants were evaluated by ELISA. **(C)** Activation markers. Peritoneal macrophages (2 × 10^6^ per well in quadruplicates) were pretreated with gp96i (3.3 μM) and then incubated with recombinant gp96 (50 pM) for 48 h. Cells were stained for surface markers. CT, untreated cells. Representative FACS dot plots, pooled results from three independent experiments. **(D)** Pancreatic islets. Freshly isolated primary mouse islets (30 per well in triplicates) were pretreated with gp96i (3.3 μM) and then incubated with recombinant gp96 (50 pM) for 48 h. Insulin release to supernatant was measured by ELISA. CT, untreated islets. **(E)** Co-culture study. Islets (30 per well in triplicates) and peritoneal macrophages (4 × 10^5^ per well) were pretreated with gp96i (3.3 μM) and then incubated with recombinant gp96 (50 pM). Forty-eight-hour later inflammatory mediators and insulin were measured in the supernatants. CT, untreated cells. Representative results from four independent experiments. Mean ± SEM, *p < 0.05, **p < 0.01.

Calibration assays were performed in order to identify the efficient concentration of gp96i that interferes with gp96 activity and also enables intact cell viability (Figure 5A). Peritoneal macrophages were exposed to the stimulatory concentration of 50 pM gp96 and varying concentrations of gp96i. According to release of nitrile and minimal change in cell viability, a concentration of 6.6 μM gp96i was chosen for subsequent studies (a stoichiometric excess of 132 inhibitor molecules per single target gp96).

In order to examine whether the inhibitor can interfere with gp96-mediated inflammatory responses in macrophages (Figures 5B,C), cells were treated with gp96i and gp96. As shown, gp96 caused an increase in release levels of IL-6 (22.6-fold) that were reduced by 79% in the presence of gp96i. Pro-inflammatory cytokines responded in a similar manner (not shown). Inducible surface levels of CD40 were similarly affected by gp96 and the introduction of gp96i (Figure 5C), in that the elevation caused by gp96 was blocked by the presence of gp96i.

Response to gp96i was also examined in primary islet cultures (Figure 5D) and in a co-culture of islets and macrophages (Figure 5E). As shown, exogenous gp96 induced the release of IL-6 from islets (9.57-fold from control), which were restored to 3.93 ± 0.04-fold from resting levels by added gp96i. A reduction in accumulated supernatant insulin exhibited in the presence of gp96 (from 2.646 to 1.628 μg/ml, mean), yet insulin levels were near-normal levels in the presence of gp96i (2.32 μg/ml). In a mixed culture, gp96 increased IL-6 release 3.91 ± 0.19-fold, but only 1.38 ± 0.08-fold in the presence of gp96i. Accumulated insulin was reduced in mixed cultures from 1.775 μg/ml in islets alone to 1.013 μg/m in islets that were cultured together with macrophages; gp96 further reduced the amount of insulin 1.42-fold in the absence of gp96i, but saw intact insulin release in the presence of gp96i.

**Figure 6:**
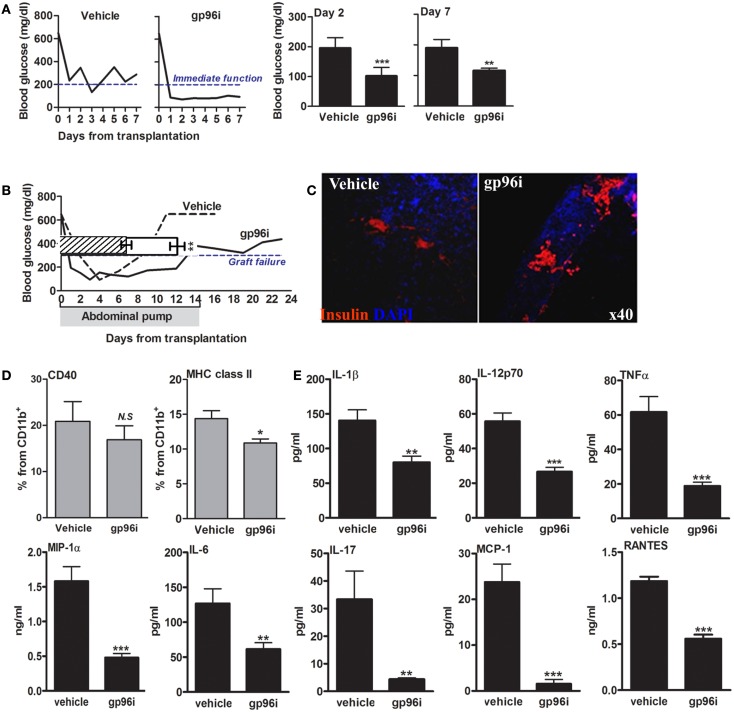
**Involvement of endogenous gp96 in islet failure during islet transplantation**. Mice were rendered hyperglycemic by single-dose STZ (225 mg/kg) and then grafted with allogeneic islets (500 islets per graft). Vehicle, untreated recipients; gp96i, gp96i-treated recipients, *n* = 8 per group. **(A)** Immediate graft function. Average of non-fasting blood glucose levels, 2 and 7 days after islet engraftment. **(B)** Prolonged graft function under continuous slow-release gp96i. Osmotic pumps were implanted in the peritoneal cavity and released gp96i in a continuous manner for 14 days. Representative glucose follow-up is shown, superimposed along the same timeline over pooled results. Shaded, the 14-day time-frame of gp96i release. **(C)** Day-7 of explanted graft immunohistochemistry. *Red*, insulin; *Blue*, DAPI nuclear counterstaining. Representative images. **(D)** Activation markers. Spleen-derived macrophages (1 × 10^6^ per sample) were stained for activation markers upon graft explanation on day 7. **(E)** Release of inflammatory mediators. Day-7 spleen-derived macrophages were cultured for 24 h and supernatants were allowed to accumulate cell release products. Inflammatory mediators in the supernatants were measured by multiplex ELISA. Mean ± SEM, **p < 0.01 and ***p < 0.001.

The function of endogenous gp96 was next challenged *in vivo* in a pancreatic islet transplantation model (Figure 6). In this model, islet function can be readily observed by following the normalization of circulating glucose. Islets from CBA/2J H-2^k^ mice were grafted under the renal capsule of hyperglycemic C57BL/6 H-2^b^ mice in the presence or absence of systemic gp96i (i.p., daily from day-1, 100 μg/mouse). As shown in Figure 6A, mice treated with gp96i exhibited superior control over glucose. To examine the function of islet grafts for 14 days, an intraperitoneal osmotic pump that released either PBS or 100 μg of gp96i every day until depleted of the inhibitor on day 14 was instilled in the peritoneal cavity. As shown in Figure 6B, gp96i-treated mice displayed superior prolongation of islet graft failure threshold after 14 days, compared to the 8-day threshold crossing in the vehicle-treated group. In a representative sample from each treatment group, grafts were explanted 2 days after graft failure and analyzed for relative insulin content by immunohistochemistry. As shown in Figure 6C, gp96i-treated mice displayed relatively larger portions of insulin-positive cells compared to vehicle-treated mice, although this parameter is not quantitative.

With focus on the immune response 7 days after islets engraftment, we examined splenocytes that were collected at that time point from each treatment group. Splenic CD11b^+^ cells were analyzed for CD40 and MHC class II surface levels (Figure 6D), as well as for inflammatory release products (Figure 6E). As shown, splenocytes collected from gp96i-treated mice exhibited a decrease in surface levels of CD40 (without reaching statistical significance) and MHC class II (*p* < 0.05) compared to splenocytes from vehicle-treated mice. A similar trend was observed in release of inflammatory mediators: splenocytes collected from gp96i-treated mice demonstrated a reduction in inflammatory mediators release (IL-1β 1.75 ± 0.06-fold, IL-6 2.06 ± 0.07-fold, IL-17 7.58 ± 0.01-fold, TNFα 3.26 ± 0.03-fold, IL-12p70 2.09 ± 0.04-fold, MCP-1 15.18 ± 0.03-fold, MIP-1α 3.57 ± 0.03-fold, and RANTES 12.59 ± 0.03-fold compared to splenocytes collected from vehicle-treated mice).

## Discussion

The beneficial effect of hAAT has been extensively reported in the context of pancreatic islet allograft transplantation and autoimmune islet damage (18–20, 22, 24, 29, 30). However, the mechanism of hAAT activity is not yet clear. The notion that hAAT may address circulating damage molecules emerged from the perplexingly high concentrations of normally circulating hAAT, particularly during the acute-phase response. We hypothesized that, as dying cells are being continually replaced by new cells, the set-point of hAAT levels may be providing protection from aberrant circulating damage molecules. Immune responses that are facilitated and enhanced by cell injury include trauma, necrosis or infection, and are primarily mediated by cell-originating DAMPs (31).

The DAMP molecule, gp96, serves as one of the most abundant chaperons in the normal cell. Several studies had proposed that the release of gp96 due to tissue damage markedly increases inflammatory responses. An example for this phenomenon can be found in RA (9, 10). Indeed, macrophages that were introduced to synovial fluid from RA patients, containing high levels of endogenous gp96, responded with an inflammatory flare. Inhibition of gp96 by specific antibodies in a murine model for RA resulted in improved tissue protection. Similarly, with no apparent known mechanism, hAAT therapy improves disease scores in a mouse model (13, 29). The overlap in animal study outcomes between gp96 inhibition and added hAAT suggest a possible relationship between these two molecules.

In the current study, we present data that suggest that during inflammatory conditions, pancreatic islets are a target of gp96-mediated inflammatory damage, and that this aspect of cell injury may be regulated by circulating hAAT. We show that gp96-treated macrophage cultures display a reduced inflammatory response and an elevated release of IL-10 in the presence of added hAAT. Macrophages isolated from hAAT-treated mice that were introduced to recombinant gp96 exhibited lower surface expression of CD40 and MHC class II, and the animals presented with reduced inflammatory serum cytokines. The effect of hAAT on gp96-mediated inflammatory responses was also evident in cultured pancreatic islets, affording gp96-exposed islets with regained insulin release. A consistent trend was observed in islets co-cultured with gp96-stimulated innate cells. Thus, islets that are presented with highly inflammatory molecular and cellular environments are injured by extracellular gp96 and protected by hAAT.

We next addressed endogenously expressed gp96. A diminishing effect of hAAT on cell-surface expression of gp96 under inflammatory conditions was observed *in vitro*. Also, in an allogeneic skin transplantation model, hAAT had decreased cell-surface expression of gp96 on macrophages that were recovered from the transplantation site. In addition, the effect of direct inhibition of endogenous gp96 by gp96i resulted in reduced inflammatory responses in macrophages, islets, and co-culture of macrophage and islets, and was demonstrated here in an islet transplantation animal model. Lastly, we show that hAAT directly binds to gp96.

Our data is supported by studies which indicate that innate immune cells respond to gp96 with inflammation (32). Inflammatory mediators released from macrophages, such as IL-1β and TNFα, play a key role in early stages of type 1 diabetes (33). In this context, the finding of elevated surface expression of CD40 and MHC class II in the presence of gp96 could contribute to macrophage interaction with and activation by T cells and enhance antigen presentation, which in turn might promote islet cell loss. In the present study, hAAT appeared to have regulated gp96-induced inflammatory responses in macrophages.

When comparing the inflammatory response mediated by gp96-stimulated macrophages *in vitro* and *in vivo*, a consistent profile emerges. Here, we utilized the commonly employed CP and ligation sepsis model, with the exception of the ligation step, so as to induce a non-lethal milder systemic inflammatory response. This allowed us to examine the effect of added exogenous gp96 without the concern of premature cessation of the cohort due to lethality. Indeed, a clear trend toward less-activated immune cells had surfaced in hAAT-treated CP-operated animals. This model was one of the first to demonstrate a clear role for DAMPs.

Although β-cell destruction in type 1 diabetes is mainly mediated by specific cytotoxic T cells and is associated with production of autoantibodies by B lymphocytes, the innate inflammatory response also serves a key role in pancreatic islet injury, and appears to be aggravated further by the presence of DAMP molecules. However, not enough data has been collected thus far regarding the sensitivity of resident immune cell populations within islets to damage signals released from injured β-cells. We addressed these aspects in the present study using mixed islets and innate cell co-cultures and observed a consistent protective effect in the presence of added hAAT. Our data thus support the notion that hAAT may be a regulator of inflammatory conditions in pancreatic islets during various injurious conditions, with a predominant capacity to blunt innate cell activation by endogenous mediators of damage.

We also addressed the appearance of gp96 on cell membranes. Cell-surface expression of gp96 under inflammatory condition was shown to correlate with tissue damage. CD14^+^ cells collected from damaged tissue demonstrated gp96 on their cell membranes, unlike cells from a non-inflamed tissue (10, 13). In a transgenic model that involves expression of cell-surface gp96 in APCs, the development of an autoimmune disease resembling SLE was shown to spontaneously evolve. In addition, in this model, resting dendritic cells expressed high levels of surface gp96 and released excess inflammatory mediators *in vitro* (12). Here, we show that hAAT reduces cell-surface expression of gp96 in BMDCs under inflammatory conditions and in peritoneal macrophages isolated from allogeneic transplantation sites. This phenomenon could be attributed to the regulatory effect of hAAT on BMDCs *in vitro*, as well as in the context of an allogeneic skin transplantation model, or it could reflect direct binding between gp96 and hAAT.

Direct interaction between gp96 and hAAT has been demonstrated in plasma samples collected from patients with type 1 diabetes (14, 34), as well as in the normal secretory pathway of intracellular hAAT (35). A similar result was illustrated in our experimental setting; gp96 pre-incubated with hAAT alone displayed loss of function; it behaved in a similar manner as gp96 that was added to cultures pretreated with hAAT. A direct interaction between gp96 and hAAT could also occur on the cell membranes, since both proteins have been shown to localize to cell-surface lipid rafts (36, 37). Although hAAT is primarily known to interact with its target proteases through a short protease-specific sequence of amino-acids, hAAT was recently shown to bind IL-8, ADAM17, and FcγRIIIb by virtue of unique bindings sites unrelated to protease inhibition (38); yet, none of the reported binding sites appears to satisfy binding conditions of hAAT to gp96 *in silico*. Further work must be performed in order to identify the domain in hAAT that interacts with gp96.

Extracellular inhibition of gp96 was examined by a polypeptide, gp96i, designed to directly interact with gp96 (26). Here, we demonstrate that gp96i reduces extracellular gp96-mediated inflammatory responses in peritoneal macrophages. The beneficial effect of gp96i was also demonstrated in a sterile pancreatic islet transplantation model. We show here for the first time that inhibition of endogenous gp96 by a specific inhibitor improves immediate islet graft function. Improved islet function could be attributed to low concentrations of IL-1β, a cytokine that is known to mediate islet injury. Recently, it has been reported that neutralizing antibodies to the DAMP molecule, high-mobility group box 1 exhibited similar outcomes to gp96 blockade (39). These data suggest that cell injury may be sensed by pancreatic islets and then become responsible for amplifying local immune cell responses, a phenomenon that may be intended to clear massive cell loss during islet transplantation. The importance of inflammation-mediating receptors, such as TLR2/4, on pancreatic islet cells post-transplantation has been demonstrated in several studies (39–41). Here, continuous treatment with gp96i by an intraperitoneal osmotic pump resulted in superior graft function, at least as long as gp96i was released from the apparatus. The precise connection between gp96 and islet damage during transplantation deserves further investigation.

Considering that hAAT provides marked benefit in multiple diabetes models, our findings provide immunological relevance to the interaction between hAAT and human gp96 reported by Finotti et al. to occur in diabetic individuals. Indeed, our data suggest that hAAT may serve as natural regulator of gp96-mediated inflammatory responses. Additionally, we suggest that gp96 may play a role in the immune response during allogeneic transplantation and that gp96 can impair immediate graft function. Taken together, this important activity of hAAT may place the clinically available molecule for consideration as an added therapeutic in pathologies that are intensified by tissue damage, such as ischemic heart disease (16), graft-versus-host disease (17), and other novel clinical indications (15).

## Conflict of Interest Statement

The authors declare that the research was conducted in the absence of any commercial or financial relationships that could be construed as a potential conflict of interest.
